# Supplementary material for: Identification of CircRNA signature associated with tumor immune infiltration to predict therapeutic efficacy of immunotherapy
Source: Nat Commun. 2023 May 3;14:2540. doi: 10.1038/s41467-023-38232-y (PMC10156742; doi:10.1038/s41467-023-38232-y)
Supplement: Supplementary file 1 — Supplementary Information [file 41467_2023_38232_MOESM1_ESM.pdf]

Supplementary Information for:

Identification of CircRNA signature associated with tumor immune infiltration to predict therapeutic efficacy of immunotherapy

Yu Dong[1,2,3,4#], Qian Gao[1,5,6#], Yong Chen[7,8#], Zhao Zhang[9,10#], Yanhua Du[2,3#],Yuan Liu[9,11,12], Guangxiong Zhang[4,5], Shengli Li[13], Gaoyang Wang[2,3], Xiang Chen[1,5*], Hong Liu[1,5*], Leng Han[9,11,12*], Youqiong Ye[2,3*]

[1]Department of Dermatology, Hunan Key Laboratory of Skin Cancer and Psoriasis, Hunan Engineering Research Center of Skin Health and Disease, Xiangya Clinical Research Center for Cancer Immunotherapy, Furong Laboratory, Changsha, Hunan, 410008, P.R. China
[2]Center for Immune-Related Diseases at Shanghai Institute of Immunology, Ruijin Hospital, Shanghai Jiao Tong University School of Medicine, Shanghai, 200025, P.R. China
[3]Shanghai Institute of Immunology, State Key Laboratory of Oncogenes and Related Genes, Department of Immunology and Microbiology, Shanghai Jiao Tong University School of Medicine, Shanghai, 200025, China.
[4]Lin gang Laboratory, Shanghai, 200025, China.
[5]National Clinical Research Center for Geriatric Disorders, Xiangya Hospital, Changsha, Hunan 410008, P.R. China
[6]Department of Clinical Laboratory, Xiangya Hospital, Central South University, Changsha, Hunan, China
[7]Department of musculoskeletal surgery, Fudan University Shanghai Cancer Center; Shanghai, 200032, PR China.
[8]Department of Oncology, Shanghai Medical College, Fudan University, Shanghai, 200032, PR China.
[9]Department of Biochemistry and Molecular Biology, McGovern Medical School at The University of Texas Health Science Center at Houston, Houston, TX 77030, USA
[10]MOE Key Laboratory of Metabolism and Molecular Medicine, School of Basic Medical Sciences, Fudan University, Shanghai, 200433, P.R. China
[11]Center for Epigenetics and Disease Prevention, Institute of Biosciences and Technology, Texas A&M University, Houston, TX, 77030, USA
[12]Department of Translational Medical Sciences, College of Medicine, Texas A&M University, Houston, TX, 77030, USA.
[13]Precision Research Center for Refractory Diseases, Institute for Clinical Research, Shanghai General Hospital, Shanghai Jiao Tong University School of Medicine (SJTU-SM), Shanghai 201620, China
[#]Denotes equal contribution

* Correspondence: hongliu1014@csu.edu.cn (H. L.), chenxiangck@126.com (X.C.),

leng.han@tamu.edu (L.H.), youqiong.ye@shsmu.edu.cn (Y.Y.)

This PDF file includes:

Supplementary Figure and Supplementary Figure Legend 1-6

Supplementary Table 1-6

Supplementary Data Legend 1-7

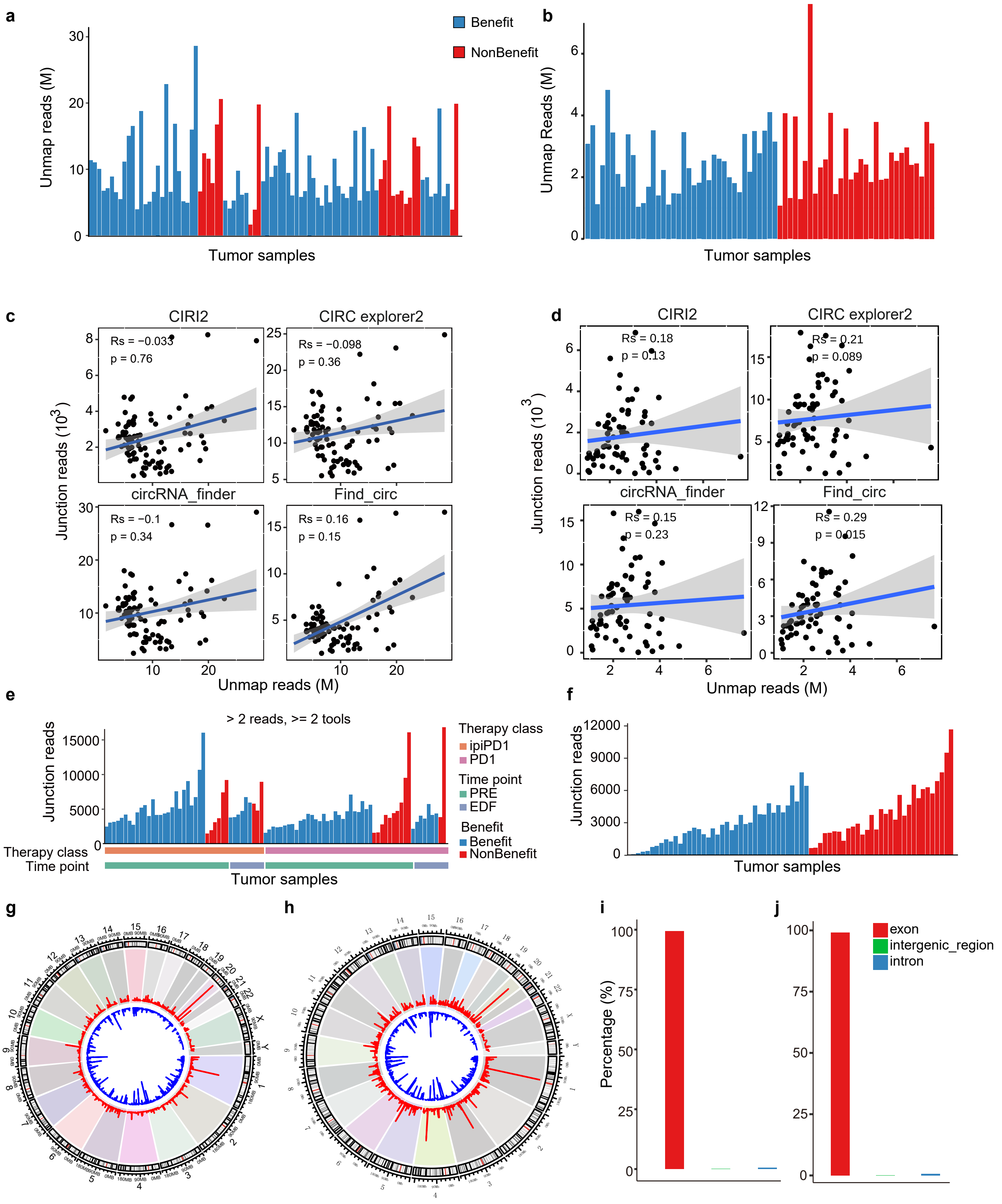

Supplementary Fig. 1 Numbers of reads and detected circRNAs in melanoma patients before and after ICB treatment in two independent cohorts. Related to Figure 1. (a-b) Number of unmapped reads for each sample in cohort 1 and cohort 2. (c-d) Two-sided Spearman's (Rs) correlation between the number of unmapped reads and the number of back-splice junction reads in each circRNA-detection tool in cohort 1 and cohort 2. The error bands in c-d indicates 95% confidence interval. (e-f) Number of back-splice junction reads for each sample in cohort 1 and cohort 2. (g-h) The distribution of benefit (red) and non-benefit (blue) related circRNAs in chromosome in cohort 1 (g) and cohort 2 (h). (i-j) Distribution of expression level of circRNAs across whole genomic regions in cohort 1 and cohort 2 (circRNAs on "+" strand were red in out circle; circRNAs on "-" strand were blue inner circle). (k-l) Genomic regions of circRNAs in cohort 1 and cohort 2 (exon, intron, and intergenic region). Source data are provided as a Source Data, Supplementary Figure 1.

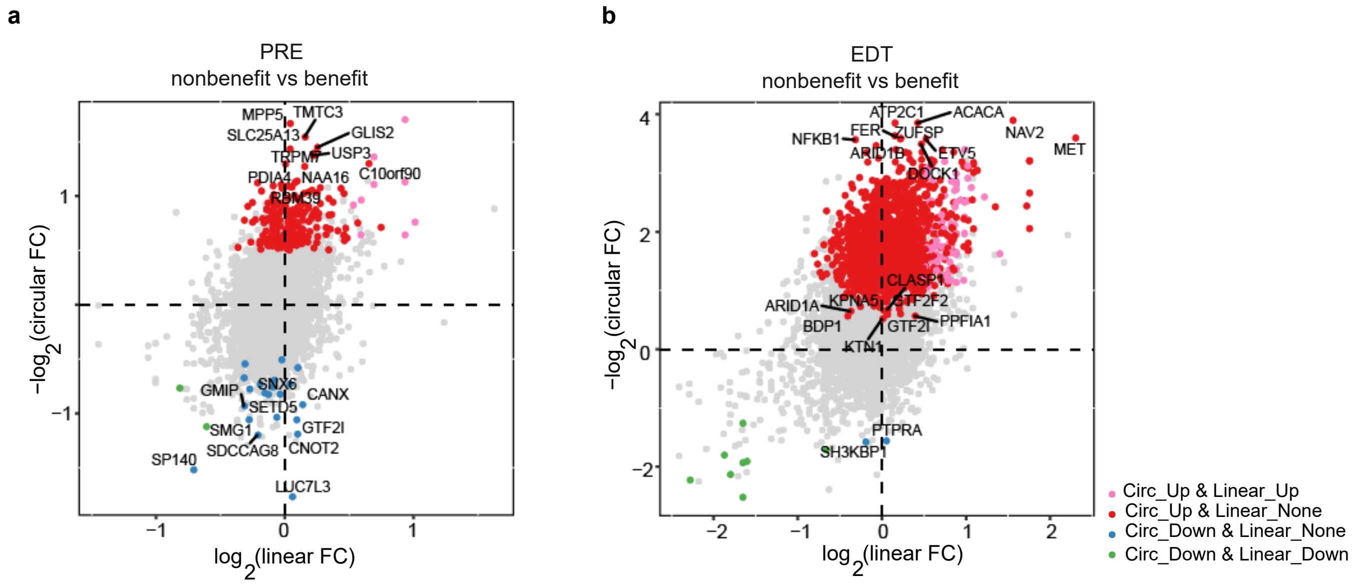

Supplementary Fig. 2 The correlation of expression change between circRNAs and linear mRNAs. Related to Figure 2. The correlation of log2 fold change of circRNA versus log2 fold change of linear mRNA expression between non-benefit (pre, n = 16; post, n = 5) and benefit (pre, n = 54; post, n = 13) melanoma samples in pre- (**a**) and post-treatment (**b**) samples respectively. Pink point were circRNAs that were upregulated and host mRNAs were also upregulated. Red point were circRNAs that were upregulated while the host mRNAs are not upregulated. Green point were circRNAs that were upregulated and host mRNAs were also upregulated. Blue point were circRNAs that were upregulated while the host mRNAs are not upregulated. Source data are provided as a Source Data, Supplementary Figure 2.

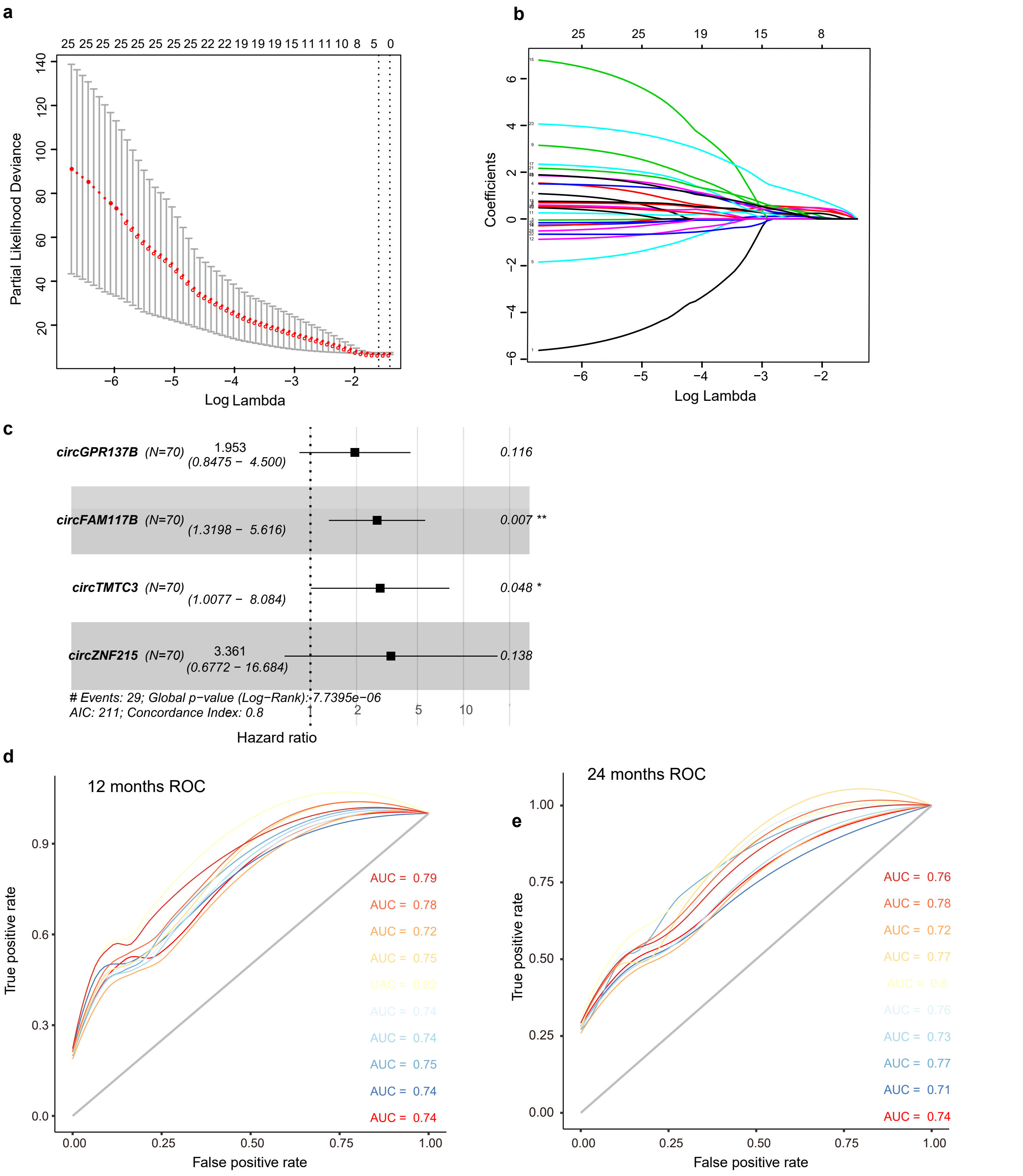

Supplementary Fig. 3 Identification of the ICBcircSig in cohort 1 and Time-dependent receiver operating characteristic (ROC) of ICBcircSig score. Related to Figure 3. (a) The partial likelihood deviance plot of LASSO Cox regression used to select candidate circRNAs. (b) LASSO coefficient profiles of candidate circRNAs. The vertical dotted lines were the optimal values by using the minimum criteria. (c) Forest plot for the hazard ratios (HRs) of multivariate Cox model of four circRNAs in the ICBcircSig score with progressive free survival (PFS). (d-e) Time-dependent receiver operating characteristic (ROC) curve at 12 and 24 months of FPS by random sampling 90% samples 10 times. Source data are provided as a Source Data, Supplementary Figure 3d-e.

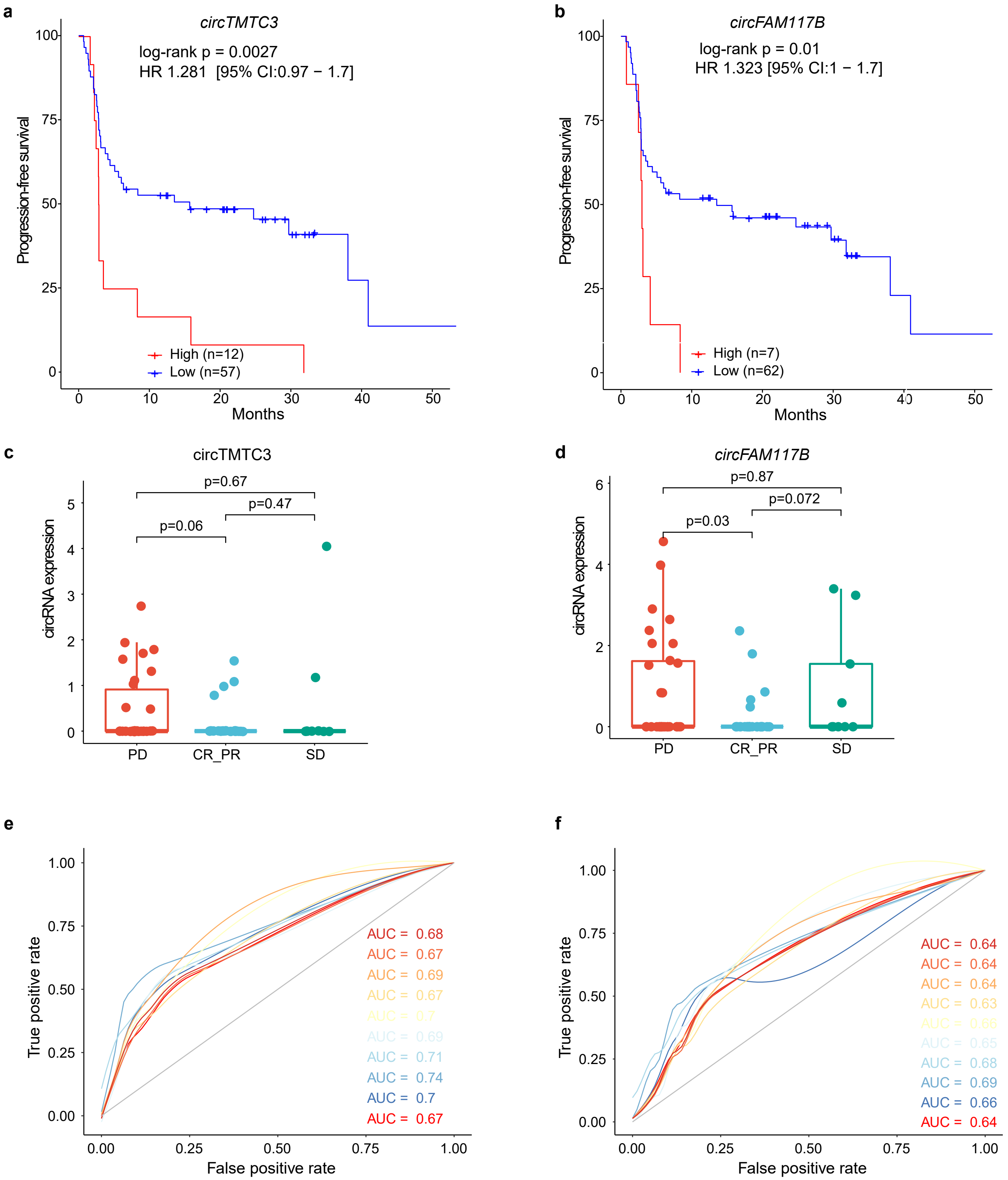

Supplementary Fig. 4 Validation of ICBcircSig score in an independent cohort. Related to Figure 4. (a-b) Kaplan–Meier survival curves of PFS between high- and low-risk patients stratified by two circRNAs using the optimal cutoff. (c-d) Expression of two circRNAs in ICBcircSig among CR/PR (n = 29), SD (n = 9), and PD (n = 30) groups. (e-f) Time-dependent receiver operating characteristic (ROC) curve at 12 and 24 months of FPS by random sampling 90% samples 10 times. Two-sided Wilcoxon rank-sum test was used in c-d. The boxes in c-d indicate the median ± 1 quartile, with the whiskers extending from the hinge to the smallest or largest value within 1.5× IQR from the box boundaries.Source data are provided as a Source Data, Supplementary Figure 4

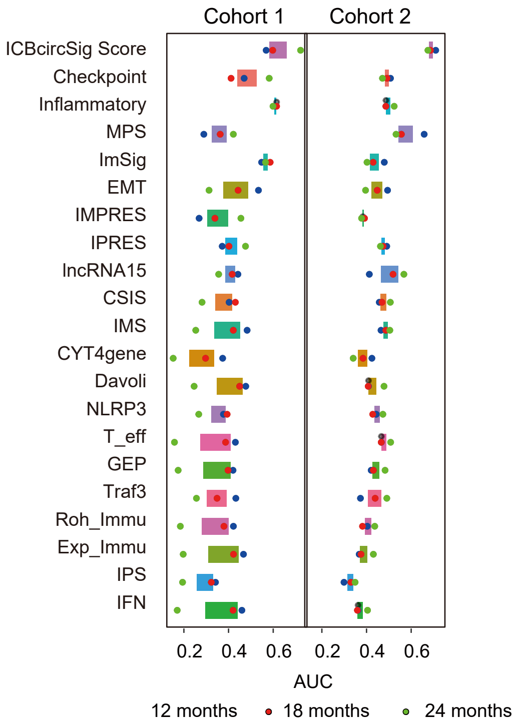

Supplementary Fig. 5 Comparative performance of ICBcircSig score and other published signatures by AUC of overall survival. Related to Figure 5. Boxplot of AUC of time-dependent ROC of 12, 18, and 24-months OS, which blue point means 12 months, red point means 18 months and green point means 24 months (n = 3). The boxes indicate the median ± 1 quartile, with the whiskers extending from the hinge to the smallest or largest value within 1.5× IQR from the box boundaries. ROC, receiver operating characteristic curve; HR, hazard ratio; AUC, Area Under the ROC Curve. Source data are provided as a Source Data, Supplementary Figure 5.

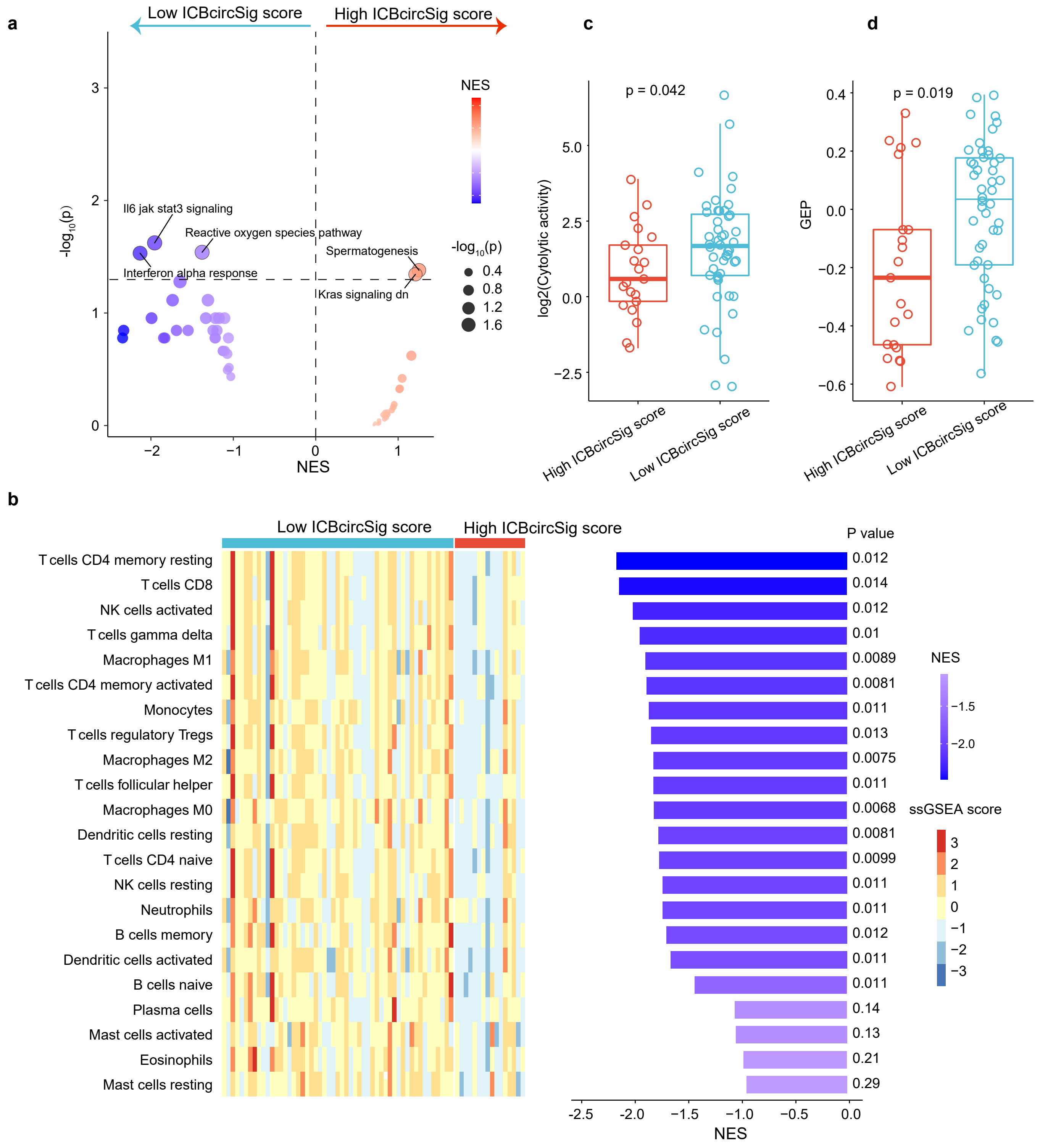

Supplementary Fig. 6 Functional characterization of the ICBcircSig score in cohort 2. Related to Figure 6. (a) Volcano plots for the enrichment of hallmarks for samples with high (n = 21) and low (n = 48) ICBcircSig score based on NES from GSEA. (b) Heatmap for ssGSEA scores by immune cell markers in groups with high (n = 21) and low (n = 48) ICBcircSig score (left panel). Enrichment of immune cell for samples with high and low ICBcircSig score based on the NES from GSEA. (c-d) Cytotoxic T cell score (c) and GEP score (d) in the high- (n = 21) and low-risk (n = 48) groups stratified by the ICBcircSig score. Two-sided Wilcoxon rank-sum test was used in c-d. The boxes in c-d indicate the median ± 1 quartile, with the whiskers extending from the hinge to the smallest or largest value within 1.5× IQR from the box boundaries.NES, Normalized Enrichment Score. GSEA, Gene Set Enrichment Analysis. Source data are provided as a Source Data, Supplementary Figure 6.

Supplementary Table 1. Clinical characteristics of ICB patients of PCR and sanger result enrolled in this study, related to Figure 3

Patient No.	Treatment	Age	Gender	Benefit	Tumor type
1	PD1	70-79	F	N	Acral
2	PD1	70-79	F	N	Acral
3	ipiPD1	60-69	M	N	Acral
4	PD1	70-79	M	N	Acral
5	PD1	60-69	M	R	Acral
6	PD1	80-89	F	R	Acral
7	PD1	50-59	F	R	Acral
8	PD1	60-69	F	R	Acral
9	PD1	60-69	M	R	Acral
10	PD1	60-69	M	R	Acral
11	PD1	50-59	F	R	Acral
12	PD1	60-69	F	R	Acral
13	PD1	50-59	F	R	Acral
14	PD1	50-59	F	R	Acral
15	PD1	40-49	M	R	Acral

Responders were defined as s with a RECIST response of progressive disease (PD), stable disease (SD), partial response (PR) and complete response (CR). Stage: TNM stage according to NCCN guideline. All patients were treated with PD-1 monoclonal antibody (PD1) or combined Anti-PD-1 + Anti-CTLA-4 therapy (ipiPD1). Abbreviation: F, female; M, male.

Supplementary Table 2. Clinical characteristics of ICB patients of in-house RNAseq set enrolled in this study , related to Figure 4 and 5

Patient No.	Treatment	Age	Gender	Biopsy Timepoint	Best RECIST response	PFS (Months)	Progressed	Stage	Tumor type
1	PD1	70-79	F	PRE	SD	2	yes	IV	Mucosal
2	ipiPD1	80-89	F	PRE	PR	17	no	IIIC	Acral
3	PD1	60-69	M	PRE	PD	15	no	IIB	Acral
4	PD1	40-49	M	PRE	SD	16	no	IIIC	Acral
5	PD1	50-59	F	PRE	PR	9	yes	IIIB	Acral
6	PD1	50-59	F	PRE	PR	6	yes	IIC	Acral
7	PD1	60-69	M	PRE	PR	11	no	IIIA	Acral
8	PD1	60-69	F	PRE	SD	20	yes	—	Mucosal
9	PD1	40-49	M	PRE	CR	17	no	—	Unknow
10	PD1	50-59	F	PRE	PR	34	no	IIIC	Acral
11	PD1	60-69	M	PRE	PR	6	yes	IIIC	Acral
12	PD1	50-59	F	PRE	PD	5	no	IV	Cutaneous
13	PD1	50-59	F	PRE	CR	2	yes	IV	Acral
14	PD1	60-69	F	PRE	SD	5	yes	IIIC	Cutaneous
15	PD1	60-69	M	PRE	CR	18	no	—	Acral
16	PD1	60-69	F	PRE	PR	6	no	IIC	Acral
17	PD1	60-69	M	PRE	PD	18	no	IIIA	Acral
18	PD1	70-79	F	PRE	PR	12	no	IIC	Acral
19	PD1	70-79	M	PRE	PD	6	yes	IV	Nodular
20	PD1	60-69	F	PRE	SD	2	yes	IV	Acral
21	PD1	60-69	F	PRE	SD	6	no	IIIA	Acral
22	PD1	70-79	F	PRE	PD	14	yes	IV	Nodular
23	PD1	50-59	F	PRE	PD	14	no	IIIA	Acral
24	PD1	50-59	M	PRE	PD	16	no	IIC	Nodular

Responders were defined as s with a RECIST response of progressive disease (PD), stable disease (SD), partial response (PR) and complete response (CR). Stage: TNM stage according to NCCN guideline. All patients were treated with PD-1 monoclonal antibody (PD1) or combined Anti-PD-1 + Anti-CTLA-4 therapy (ipiPD1). Abbreviation: F, female; M, male.

Supplementary Table 3. ICB response-related signatures, related to Figure 5

Signature name	Title of source paper	Description	PMID
lncRNA-15	Identification of 15 lncRNAs Signature for Predicting Survival Benefit of Advanced Melanoma Patients Treated with Anti-PD-1 Monotherapy	Weighted sum of the 15 gene expression levels	33922038
checkpoint	A novel immune checkpoint-related seven-gene signature for predicting prognosis and immunotherapy response in melanoma	Weighted sum of the 7 gene expression levels	32731180
IMS	Ratio of the interferon-γ signature to the immunosuppression signature predicts anti-PD-1 therapy response in melanoma	log2 transform and housekeeping normalised gene expression, then arithmetic mean to calculate two score(6 IFN and 18 IMS) , final score is the difference between two score	33542239
IPRES	Genomic and transcriptomic features of response to anti-PD-1 therapy in metastatic melanoma/Gene Signatures of Tumor Inflammation and Epithelial-to-Mesenchymal Transition (EMT) Predict Responses to Immune Checkpoint Blockade in Lung Cancer with High Accuracy	Summing the log2 Z scores of 16 genes	31683225
IFN-γ	IFN-gg–related mRNA profile predicts clinical response to PD-1 blockade	Mean of the 6 gene expression levels	28650338
Exp. Immu.	IFN-gg–related mRNA profile predicts clinical response to PD-1 blockade	Mean of the 18 gene expression levels	28650338
Roh Immu	Integrated molecular analysis of tumor biopsies on sequential CTLA-4 and PD-1 blockade reveals markers of response and resistance	Geometric mean of the 41 gene expression levels	28251903
IMPRES	Robust prediction of response to immune checkpoint blockade therapy in metastatic melanoma	The Fi,j(x) over the 15 IMPRES checkpoint pairs (features). This leads to a binary vector of length 15 for each sample. The total number of ‘1’s in this vector denotes the sample’s IMPRES score (ranging between 0 and 15).	30127394
T eff.	Atezolizumab versus docetaxel for patients with previously treated non-small-cell lung cancer (POPLAR): a multicentre, open-label, phase 2 randomised controlled trial	Mean of the 8 gene expression levels	26970723
Davoli	Tumor aneuploidy correlates with markers of immune evasion and with reduced response to immunotherapy	Mean of the 7 gene expression levels	28104840
GEP	IFN-gg–related mRNA profile predicts clinicalresponse to PD-1 blockade	ssGSEA of the 18 gene expression levels	28650338
CYT	Molecular and genetic properties of tumors associated with local immune cytolytic activity	Geometric mean of the 4 gene expression levels	25594174
IPS	Pan-cancer Immunogenomic Analyses Reveal Genotype-Immunophenotype Relationships and Predictors of Response to Checkpoint Blockade	Sum of four type of score and mapping to discreet score	28052254
NLRP3	Pan-cancer analysis of NLRP3 inflammasome with potential implications in prognosis and immunotherapy in human	ssGSEA of the 30 gene expression levels	33212483
MPS	Multimodel preclinical platform predicts clinical response of melanoma to immunotherapy	Weighted sum of the 45 gene expression levels	32284588
CSIS	Cancer-Specific Immune Prognostic Signature in Solid Tumors and Its Relation to Immune Checkpoint Therapies	Weighted sum of the 30 gene expression levels	32882873
ImSig	A gene expression signature of TREM2hi macrophages and γ δ T cells predicts immunotherapy response	Summing the log2 Z scores of 108 genes	33033253
Inflammatory	Gene Signatures of Tumor Inflammation and Epithelial-to-Mesenchymal Transition (EMT) Predict Responses to Immune Checkpoint Blockade in Lung Cancer with High Accuracy	Summing the log2 Z scores of 27 genes	31683225
EMT	Gene Signatures of Tumor Inflammation and Epithelial-to-Mesenchymal Transition (EMT) Predict Responses to Immune Checkpoint Blockade in Lung Cancer with High Accuracy	Sum of the log2 Z scores of 6 established mesenchymal genes subtracting the sum of the log2 Z scores of 6 established epithelial genes	31683225
Traf3	Therapeutically Increasing MHC-I Expression Potentiates Immune Checkpoint Blockade	Weighted sum of the 400 gene expression levels	33589424

Supplementary Data Legend

Supplementary Data 1

Clinical characteristics of ICB patients of training set enrolled in this study, related to Figure 1.

Supplementary Data 2

Clinical characteristics of ICB patients of testing set enrolled in this study, related to Figure 1.

Supplementary Data 3

The circRNA expression of cohort 1.

Supplementary Data 4

The circRNA expression of cohort 2.

Supplementary Table 5

Differentially expressed genes between non-responders and responders, related to Figure 2.

Supplementary Data 6

ICB response-associated circRNA-miRNA-mRNA interactions, related to Figure 2.

Supplementary Data 7

The circRNA expression of in-house cohort 3.
