# Supplementary material for: Identification of CircRNA signature associated with tumor immune infiltration to predict therapeutic efficacy of immunotherapy
Source: Nat Commun. 2023 May 3;14:2540. doi: 10.1038/s41467-023-38232-y (PMC10156742; doi:10.1038/s41467-023-38232-y)
Supplement: Supplementary file 3 — Reporting Summary [file 41467_2023_38232_MOESM3_ESM.pdf]

nature portfolio

Corresponding author(s): Hong Liu, Xiang Chen, Leng Han, Youqiong Ye

Last updated by author(s): Mar 18, 2023

Reporting Summary

Nature Portfolio wishes to improve the reproducibility of the work that we publish. This form provides structure for consistency and transparency in reporting. For further information on Nature Portfolio policies, see our Editorial Policies and the Editorial Policy Checklist.

Statistics

For all statistical analyses, confirm that the following items are present in the figure legend, table legend, main text, or Methods section.

n/a	Confirmed	
[ ]	[x]	The exact sample size (*n*) for each experimental group/condition, given as a discrete number and unit of measurement
[ ]	[x]	A statement on whether measurements were taken from distinct samples or whether the same sample was measured repeatedly
[ ]	[x]	The statistical test(s) used AND whether they are one- or two-sided<br>*Only common tests should be described solely by name; describe more complex techniques in the Methods section.*
[ ]	[x]	A description of all covariates tested
[ ]	[x]	A description of any assumptions or corrections, such as tests of normality and adjustment for multiple comparisons
[ ]	[x]	A full description of the statistical parameters including central tendency (e.g. means) or other basic estimates (e.g. regression coefficient) AND variation (e.g. standard deviation) or associated estimates of uncertainty (e.g. confidence intervals)
[ ]	[x]	For null hypothesis testing, the test statistic (e.g. *F*, *t*, *r*) with confidence intervals, effect sizes, degrees of freedom and *P* value noted<br>*Give P values as exact values whenever suitable.*
[x]	[ ]	For Bayesian analysis, information on the choice of priors and Markov chain Monte Carlo settings
[x]	[ ]	For hierarchical and complex designs, identification of the appropriate level for tests and full reporting of outcomes
[ ]	[x]	Estimates of effect sizes (e.g. Cohen's *d*, Pearson's *r*), indicating how they were calculated

Our web collection on statistics for biologists contains articles on many of the points above.

Software and code

Policy information about availability of computer code

Data collection	No software was used for data collection, as publicly available datasets were used.
Data analysis	Codes were implemented in R 3.6.0 and are deposited in https://github.com/Yelab2020/ICBcircSig.

For manuscripts utilizing custom algorithms or software that are central to the research but not yet described in published literature, software must be made available to editors and reviewers. We strongly encourage code deposition in a community repository (e.g. GitHub). See the Nature Portfolio guidelines for submitting code & software for further information.

Data

Policy information about availability of data

All manuscripts must include a data availability statement. This statement should provide the following information, where applicable:

- Accession codes, unique identifiers, or web links for publicly available datasets
- A description of any restrictions on data availability
- For clinical datasets or third party data, please ensure that the statement adheres to our policy

The raw data of bulk RNA-seq generated in this study have been deposited in the Genome Sequence Archive (GSA) under accession code HRA003368. The raw data of bulk RNA-seq data are available under restricted access for human resource, access can be obtained by requesting and following the guidelines for GSA for non-commercial use at https://ngdc.cncb.ac.cn/gsa-human/request/ HRA003368. The raw data of cohort 1 and 2 were downloaded from the European Nucleotide Archive (ENA, PRJEB23709) and the database of Genotypes and Phenotypes (dbGaP, phs000452.v3.p1). The processed gene expression data of cohort 1, cohort 2, and in-house cohort were submitted as Supplementary Data 1, 2, and 4. The data information (e.g., sample size, overall survival times, progressive free survival

time) of cohort 1, cohort 2, and in-house cohort were summarized in Supplementary Table 1, 2, and 5. The sequence information of miRNAs of human were download from miBase (https://www.mirbase.org/). The miRNA–mRNA pairs were obtained from Tarbase (http://www.microrna.gr/tarbase) and TargetScan (https://www.targetscan.org/) database. Source data are provided with this paper.

Human research participants

Policy information about studies involving human research participants and Sex and Gender in Research.

Reporting on sex and gender	1. 9 (60%) out of 15 melanoma patients are female for qRT-PCR validation in this study, no sex or gender analysis was carried out.<br>2. Since Sanger sequencing validate expression and junction of circRNAs, no sex or gender analysis was carried out.<br>3. 15 (62.5%) out of 24 melanoma patients with RNA-seq are female for ICBcircSig score model validation in this study. The ICBcircSig score model does not involved the gender variable, therefore no sex or gender analysis was carried out.
Population characteristics	1. The cohort of 15 patients with stage III or IV melanoma evaluated in this study received anti-PD-1 treatment after the failure of other therapies and were collected between May 2018 and September 2020. They were treated with anti-PD-1 monotherapy (n = 11 pembrolizumab 200 mg/cycle every 3 weeks; n = 3 Toripalimab 200 mg/cycle every 2 weeks; n=1 Camrelizumab 200 mg/cycle every 2 weeks). The median age of the patients was 64 years (range, 43 to 85 years), with 6 (40%) male patients and 9 (60%) female patients.<br>2. The cohort of 24 patients with melanoma evaluated in this study received anti-PD-1 treatment or combination anti-PD-1 and anti-CTLA-4 were collected between May 2018 and September 2020. They were treated with anti-PD-1 monotherapy (n = 23 pembrolizumab 200 mg/cycle every 3 weeks; n=1 ipilimumab 200 mg/cycle every 2 weeks). The median age of the patients was 62.5 years (range, 41 to 85 years), with 9 (37.5 %) male patients and 15 (62.5%) female patients. The clinical information is summarized in Supplementary Table 6.
Recruitment	Our study does not involve patient recruitment. Patients with tissues before PD-1 Ab treatment, and clinicopathological information were retrospectively retrieved.
Ethics oversight	The study was conducted in accordance with ethical guidelines of U.S. Common Rule. All tissue samples were collected in compliance with the informed consent policy. The study protocol was approved by the Institutional Review Board of Fudan University Shanghai Cancer Center (1906203-3).

Note that full information on the approval of the study protocol must also be provided in the manuscript.

Field-specific reporting

Please select the one below that is the best fit for your research. If you are not sure, read the appropriate sections before making your selection.

☒ Life sciences ☐ Behavioural & social sciences ☐ Ecological, evolutionary & environmental sciences

For a reference copy of the document with all sections, see nature.com/documents/nr-reporting-summary-flat.pdf

Life sciences study design

All studies must disclose on these points even when the disclosure is negative.

Sample size	No sample size calculation was performed. We followed the routine biological replicate requirement in the field, n >= 3 for each group.
Data exclusions	We collected comparable number of ICB treated cancer patients in two cohorts. For cohort2 , patients whose biopsy site is not skin, incomplete demographic and follow-up information and biopsy time for after-treatment were excluded.
Replication	All experiments were performed independently at least three times using biologically independent replicates. All replication attempts were successful.
Randomization	Sequencing data of cohort 1 and 2 were obtained from public data resources and sequencing data of in-house cohort 3 is retrospective cohort , so randomization is not relevant to our study.
Blinding	The investigators were blinded to group allocations during data collection and/or analysis.

Reporting for specific materials, systems and methods

We require information from authors about some types of materials, experimental systems and methods used in many studies. Here, indicate whether each material, system or method listed is relevant to your study. If you are not sure if a list item applies to your research, read the appropriate section before selecting a response.

Materials & experimental systems

n/a	Involved in the study
x	Antibodies
	x Eukaryotic cell lines
x	Palaeontology and archaeology
x	Animals and other organisms
x	Clinical data
x	Dual use research of concern

Methods

n/a	Involved in the study
x	ChIP-seq
x	Flow cytometry
x	MRI-based neuroimaging

Eukaryotic cell lines

Policy information about cell lines and Sex and Gender in Research

Cell line source(s)	SK-MEL-28 melanoma cell line was from American Type Culture Collection (ATCC)
Authentication	The cells were authenticated based on the morphology under microscope and growth rate.
Mycoplasma contamination	Cell lines were tested negative for mycoplasma contamination
Commonly misidentified lines (See ICLAC register)	No misidentified lines were used
