# Supplementary material for: Identification of CircRNA signature associated with tumor immune infiltration to predict therapeutic efficacy of immunotherapy
Source: Nat Commun. 2023 May 3;14:2540. doi: 10.1038/s41467-023-38232-y (PMC10156742; doi:10.1038/s41467-023-38232-y)
Supplement: Supplementary file 4 — Description of Additional Supplementary Files [file 41467_2023_38232_MOESM4_ESM.docx]

**Description of Additional Supplementary Files**

Supplementary Data 1

Description: Clinical characteristics of ICB patients of training set enrolled in this study, related to Figure 1.

Supplementary Data 2

Description: Clinical characteristics of ICB patients of testing set enrolled in this study, related to Figure 1.

Supplementary Data 3

Description: The circRNA expression of cohort 1.

Supplementary Data 4

Description: The circRNA expression of cohort 2.

Supplementary Data 5

Description: Differentially expressed genes between non-responders and responders, related to Figure 2.

Supplementary Data 6

Description: ICB response-associated circRNA-miRNA-mRNA interactions, related to Figure 2.

Supplementary Data 7

Description: The circRNA expression of in-house cohort 3.
